# AGEN: Adaptive Error Control-Driven Cross-View Geo-Localization Under Extreme Weather Conditions

**DOI:** 10.3390/s25123749

**Published:** 2025-06-15

**Authors:** Mengmeng Xu, Hongxiang Lv, Hai Zhu, Enlai Dong, Fei Wu

**Affiliations:** Faculty of Electrical and Electronic Engineering, Shanghai University of Engineering Science, Shanghai 201620, China; m320123403@sues.edu.cn (M.X.); m325122241@sues.edu.cn (H.L.); m325123302@sues.edu.cn (E.D.); fei_wu1@163.com (F.W.)

**Keywords:** geo-localization, Siamese network, DINOv2, fuzzy PID

## Abstract

Cross-view geo-localization is a task of matching the same geographic image from different views, e.g., drone and satellite. Due to its GPS-free advantage, cross-view geo-localization is gaining increasing research interest, especially in drone-based localization and navigation applications. In order to guarantee system accuracy, existing methods mainly focused on image augmentation and denoising while still facing performance degradation when extreme weather conditions are considered. In this paper, we propose a robust end-to-end image retrieval framework, AGEN, serving for cross-view geo-localization under extreme weather conditions. Inspired by the strengths of the DINOv2 network, particularly its strong performance in global feature extraction, while acknowledging its limitations in capturing fine-grained details, we integrate the DINOv2 network with the Local Pattern Network (LPN) algorithm module to extract valuable classification features more efficiently. Additionally, to further enhance model robustness, we innovatively introduce an Adaptive Error Control (AEC) module based on fuzzy control to optimize the loss function dynamically. Specifically, by adjusting loss weights adaptively, the AEC module allows the model to better handle complex and challenging scenarios. Experimental results demonstrate that AGEN achieves a Recall@1 accuracy of 91.71% on the University160k-WX dataset under extreme weather conditions. Through extensive experiments on two well-known public datasets, i.e., University-1652 and SUES-200, AGEN achieves state-of-the-art Recall@1 accuracy in both drone-view target localization tasks and drone navigation tasks, outperforming existing models. In particular, on the University-1652 dataset, AGEN reaches 95.43% Recall@1 in the drone-view target localization task, showcasing its superior capability in handling challenging scenarios.

## 1. Introduction

Cross-view geo-localization refers to the process of matching a given image (typically a ground-level or drone-captured image) with a geo-tagged reference satellite image. This process can be viewed as a retrieval task, where the goal is to identify and locate the satellite image most relevant to the given image. This technology has a wide range of applications in several fields, including target detection, environmental monitoring and management, precision agriculture, military reconnaissance, intelligence gathering, target tracking, and even the monitoring of archaeological sites and cultural heritage sites [1,2,3,4]. Cross-view geo-localization, when combined with an inertial measurement unit (IMU), also holds significant application potential, while ground-view geo-localization provides precise positioning for autonomous vehicles, and drone-view geo-localization enhances the attitude control (i.e., pitch, yaw, and roll stabilization), navigation accuracy, and autonomous flight capabilities of drones [5], especially promising for high-precision localization and path planning in environments with weak or absent GPS signals [6].

Due to great spatial and temporal differences, e.g., varying angles and fields of view, between satellite and ground-view images, the matching task is very challenging in dealing with significant disparities in scale, perspective, and background [7,8]. On the other side, with the continuous development of drone technology, drones have become more convenient and cost-effective for image acquisition in recent years. Compared with traditional ground-based imagery, the angular difference between the drone view and satellite view is smaller, and there are fewer obstacles, such as trees and surrounding environments, in the images captured by drones. These advantages make drone-view images more suitable for cross-view matching tasks [9,10].

With the rapid development of deep learning, significant progress has been made in cross-view geo-localization technology [11,12]. However, in practical applications, drone-based cross-view geo-localization still faces numerous challenges, particularly due to factors such as viewpoint, illumination, resolution, and seasonal variations. Specifically, existing research has shown that extreme weather conditions, such as heavy rain and snow, will lead to a significant decline in cross-view geo-localization performance [13].

To address the impact of extreme weather, traditional solutions typically focused on first denoising the images affected by weather noise before performing cross-view geo-localization. Weather noise removal methods can be categorized into single-weather and multi-weather denoising approaches. Single-weather denoising methods, such as those proposed by Ren et al. [14] and Jiang et al. [15], primarily focused on removing specific weather noise (e.g., snow and raindrops). The DID-MDN network proposed by Zhang and Patel [16] and the dual attention mechanism introduced by Quan et al. [17] also successfully removed raindrop noise. However, these methods are typically limited to specific weather noise and struggle to effectively handle varying weather conditions, particularly in complex weather environments where their performance is constrained.

**Figure 1:**
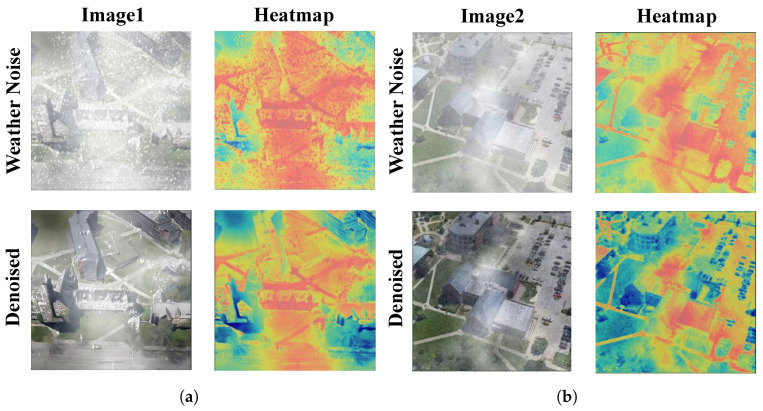
(**a**,**b**) each compare the heatmaps of noisy images from University160K-WX and their denoised counterparts.

For multi-weather noise removal, CRNet [18] and DSANet [19] effectively removed various types of weather noise, such as fog, snow, and rain, through multi-branch modules and domain attention mechanisms. The adversarial learning approach proposed by Li et al. [20] can handle multiple severe weather conditions, while Chen et al. [21] improved denoising efficiency through a two-stage knowledge-learning process. Despite these advancements in noise removal, these methods often result in the loss of key image features, as shown in Figure 1, which affects the accuracy of cross-view geo-localization [22]. The multi-environment adaptive network proposed by Wang et al. [23] adjusted domain transfer adaptively based on environmental style information, achieving good experimental results, particularly excelling across multiple datasets. However, this method still has significant limitations when dealing with extreme weather conditions in real-world scenarios and has not fully addressed the problems in practical applications.

In summary, existing image denoising and cross-view geo-localization methods have made significant progress, particularly in handling images under multi-weather conditions. They still face great challenges, especially in handling images with severe weather noise; the optimization and enhancement of deep learning frameworks remains an imperative and unsolved problem.

To address the challenges, this paper proposes a twin network model, AGEN, which fully leverages the advantages of DINOv2 [24], especially its strong global feature extraction capabilities. However, DINOv2 exhibits certain limitations in capturing fine-grained features. To overcome this, the Local Pattern Network (LPN) [25] is creatively integrated, effectively compensating for DINOv2’s shortcomings in fine-grained feature extraction. Additionally, we innovatively propose the fuzzy PID control-based Adaptive Error Control (AEC) module. The AEC module dynamically adjusts loss weights by incorporating historical error information and its temporal evolution, aiming to mitigate training instability caused by extreme weather conditions. By enabling stage-aware optimization throughout the training process, the AEC module enhances the model’s robustness and convergence under severe environmental variations. Furthermore, we employ a data enhancement algorithm for better model training, which applies nine different weather conditions to the drone images in the University-1652 dataset [26]. Our main contributions are summarized as follows.

- We propose a twin network AGEN with shared weights for cross-view geo-localization under extreme weather conditions, which effectively employs DINOv2 as the backbone network and integrates LPN for more comprehensive feature information extraction and matching.
- The innovative fuzzy control-based AEC module is proposed to dynamically adjust the loss weights and optimize the learning process of the model according to the historical errors and their trends so that the model can adaptively learn the knowledge at different training stages. To further guarantee the robustness of AGEN under various weather conditions, we also extend the original dataset by 9 times for model training.
- To validate the effectiveness of AGEN, we conduct extensive experiments. On the University-1652 dataset, AGEN achieves an impressive Recall@1 accuracy of 95.43% on the drone-view target localization task and 96.72% accuracy on the drone navigation task, outperforming other state-of-the-art models. Under extreme weather conditions on the University160k-WX dataset, the AGEN model delivers remarkable results with a Recall@1 of 91.62%, and it also performs well on the SUES-200 dataset. These results highlight the model’s robustness and effectiveness in handling complex environmental conditions.

The organization of this paper is as follows: Section 2 reviews the latest advancements in cross-view geo-localization, explores the application of modern backbone networks in scene matching, and summarizes key trends in the development of loss functions. Section 3 provides a detailed introduction to our proposed framework, including dataset extension, the integration of DINOv2, the role of the LPN module in fine-grained feature extraction, and the implementation of the AEC module for loss optimization. Section 4 presents an in-depth analysis of our experiments, covering dataset characteristics, implementation strategies, comparisons with state-of-the-art methods, and ablation studies. Section 5 discusses the limitations of the proposed approach and outlines potential directions for future improvement. Finally, Section 6 summarizes the research findings and outlines directions for future work.

## 2. Related Work

### 2.1. Geo-Localization Datasets and Methods

Drones play a crucial role in cross-view geo-localization research, particularly in the matching of drone and satellite images, which has become a hot topic in current research. Addressing this issue is of significant practical value and theoretical importance for enhancing the accuracy and robustness of cross-view geo-localization.

With the compelling development of deep learning technology, data becomes an indispensable part of any image processing area. The University-1652 dataset [26] played a pioneering role in multi-view, multi-source geo-localization research, containing images of 1652 university buildings and laying the foundation for the early development of the field. Building upon this, the SUES-200 dataset [27] expanded the research scope by collecting 24,120 real drone images and their corresponding satellite images, significantly improving the generalization capability of models. To address the impact of extreme weather conditions on geo-localization accuracy, the University160k-WX dataset [13] was specifically created, generating images with weather noise such as fog, rain, snow, and other adverse weather conditions to simulate complex real-world environments. Together, these datasets provide an important testing platform for evaluating model performance in maintaining geo-localization robustness under extreme weather conditions. Additionally, we further expand the original University-1652 dataset for better model evaluation.

Previous geo-localization research primarily focused on extracting local and global features from images to improve scene-matching accuracy. For example, [28] emphasized the fine-grained features of orientation information and proposed two granularity methods to enhance the orientation estimation of street view images, which effectively improved geo-localization performance. In recent years, feature mining has gradually shifted towards higher-density feature extraction. FSRA [29] introduced a feature segmentation and region alignment method, which enhanced the model’s ability to understand contextual information and instance distribution. Conversely, PCL [9] transformed drone image viewpoints into satellite viewpoints through perspective projection. This approach, combined with satellite viewpoint labeling, alleviated the learning burden on the network while effectively utilizing contextual information.

Local-Pattern Network (LPN) [25] introduced a partitioning strategy to train the model for finer-grained feature extraction, leading to significant performance improvements on the University-1652 dataset. Multiple Granularity Network [30] further divided the image into multiple strips and extracted varying granularities of information from different local branches, thereby combining global and local features, which significantly improved multi-granularity feature matching. To address the issue of combining global and local features, TransGeo [31] adopted the Transformer architecture for global information modeling and improved model performance through the attention mechanism. Self-Attention Image Generator [32] enhanced image generation and global information modeling using the self-attention mechanism, thus improving the model’s performance on complex tasks. To address the image distortion problem, SeGCN [33] proposed a semantic inference model that divided global features into multiple local features, achieving better results across multiple datasets. SRLN (Scale-aware Radial Local Network) [34] proposed a multi-scale feature fusion network consisting mainly of a Radial-Slicer Network and LPN. This structure verified the effectiveness of LPN while focusing on fine-grained features of orientation and global environmental characteristics.

Inspired by the above studies, we innovatively incorporated LPN into the AGEN framework, addressing the framework’s limitations in fine-grained feature extraction. This enables AGEN to more accurately capture detailed local information under complex weather conditions, thereby achieving higher accuracy and stability in scene-matching tasks under extreme weather.

### 2.2. Modern Backbones

In visual tasks, while effective at extracting local features, traditional convolutional neural networks [35] faced challenges in capturing long-range dependencies. To overcome this limitation, the attention mechanism [36] was introduced, significantly enhancing the model’s ability to capture global information by focusing on the relationships between different regions in an image. Building on this, the Vision Transformer (ViT) [37] further divided images into patches and leveraged the self-attention mechanism to capture long-distance dependencies, which greatly improved the model’s expressive power. However, ViT had high data and computational requirements, leading to unstable performance, especially on small datasets.

To address these challenges, the Swin Transformer [38] introduced a hierarchical local-window self-attention mechanism. This approach divided the image into smaller windows, reducing computational complexity while maintaining a hierarchical feature structure. Although this approach improved computational efficiency and made Swin Transformer applicable to a variety of visual tasks, it still exhibited limitations in cross-view tasks. In response to these limitations, multimodal models such as CLIP [39] were proposed, which combined image and text information to train cross-modal features. EVA [40] and EVA02 [41] further optimized multimodal feature representations, enhancing model adaptability and task performance through knowledge distillation techniques [42].

However, these models primarily relied on supervised data, which limited their ability to effectively leverage unlabeled data. Inspired by the success of BERT [43], DINO [44], and iBOT [45], self-supervised learning was introduced. This approach enabled models to learn semantic representations in an unsupervised manner through masked prediction. DINO employed a self-distillation technique, which allowed it to derive more robust features. On the other hand, iBOT improved the accuracy of feature learning by utilizing dynamic online encoders. Building upon this foundation, DINOv2 [24] combined the strengths of DINO and iBOT. It incorporated optimized data processing and architecture, which enhanced the stability and performance of the model when applied to large-scale datasets. As a result, DINOv2 contributed to the advancement of unsupervised learning for visual tasks. The Siamese Network [46], a similarity-based architecture, demonstrated excellent performance in image-matching tasks. By employing a dual-network structure with shared parameters, the Siamese network efficiently computed the similarity between input images. This approach proved particularly effective in handling image matching across varying viewpoints, such as in drone and satellite image matching.

Inspired by the strengths of DINOv2 in global feature extraction, this paper proposes the AGEN framework, which fully leverages DINOv2’s robust capability in capturing long-range dependencies, providing a solid foundation for handling large-scale visual tasks.

### 2.3. Development of the Loss Function

In computer vision tasks, the choice and optimization of the loss function are crucial for model performance. The cross-entropy loss function [47] is widely used in classification tasks, but it is sensitive to noise and outliers, and in cases of class imbalance, it often leads to model bias toward the majority class. To address this issue, researchers proposed the weighted cross-entropy loss function [48,49], which assigned higher weights to minority class samples, reducing the model’s bias toward the majority class and significantly improving performance in class-imbalanced tasks.

As task complexity increases, particularly in similarity learning and matching tasks, the traditional cross-entropy loss becomes increasingly ineffective. To enhance the model’s discriminative ability, the triplet loss function [50] was introduced. It minimized the distance between the anchor and the positive sample while maximizing the distance between the anchor and the negative sample, thereby promoting the learning of discriminative features. However, triplet loss incurs high computational complexity, especially for large-scale datasets, and the sample selection process poses a significant challenge. To address this, angular triplet loss [51] was proposed, which introduced angular information into the distance calculation, focusing on the angular differences between samples and enhancing the model’s sensitivity to subtle feature space differences while also reducing computational complexity.

Despite its success in metric learning, triplet loss has limitations in pairwise similarity tasks. To address this, contrastive loss [52] was introduced, which optimized feature discrimination by bringing similar samples closer and pushing dissimilar samples farther apart. However, traditional contrastive loss struggles to capture subtle class differences in multi-class tasks. To overcome this, researchers proposed improvements such as the Edge Stereo network [53] and the InfoCD method [54], which introduced edge-aware loss and an improved Chamfer distance for contrastive learning, optimizing edge accuracy in matching tasks. Additionally, circle loss [55] ensured more compact distributions of features from different classes in the feature space, further enhancing performance in fine-grained classification and face recognition tasks.

Although these improved loss functions have made significant advancements in matching accuracy and multi-class task performance, they still rely on static or pre-defined loss strategies, lacking dynamic feedback mechanisms. As a result, they struggle to adapt to dynamic scenes, limiting the model’s generalization ability and robustness.

Inspired by [56], to overcome existing limitations, we design an AEC module, which incorporates fuzzy PID control methods into cross-view scene matching tasks. The AEC module dynamically weights the cross-entropy loss function and provides real-time feedback on error changes, optimizing the learning transfer process of the student network.

## 3. Methods

### 3.1. Overall Architecture

**Figure 2:**
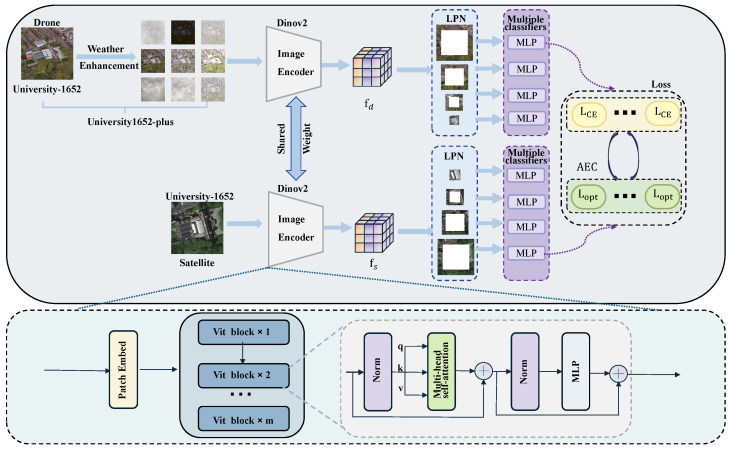
Network architecture diagram of AGEN.

The overall framework of our solution is depicted in Figure 2. First, we propose a dataset expansion and augmentation method to address the challenges of cross-view matching under extreme weather conditions. Second, we innovatively adopt DINOv2 as the backbone network for feature extraction and feed its deep-level features into the Local-Pattern Network (LPN) and multi-branch classification heads to capture more fine-grained pattern features. Finally, we introduce a loss function optimization module based on fuzzy control, aiming to adaptively adjust the cross-entropy loss function.

### 3.2. Dataset Expansion

**Figure 3:**
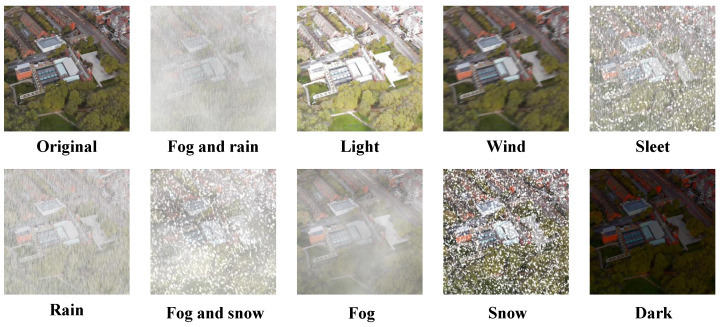
Weather conditions considered in University1652-plus.

This section outlines the process and rationale for creating our augmented dataset, University1652-plus. In order to enhance the robustness of cross-view geo-localization models under adverse weather conditions, the University1652-plus dataset is developed by extensively expanding the original University-1652 dataset. Specifically, nine distinct weather conditions are selected to simulate various challenging environments for drone-view images, including dark, rain, fog, snow, fog and rain, fog and snow, sleet, light, and wind, as shown in Figure 3. These selections aim to cover a wide range of weather scenarios that could affect drone images.

The original University-1652 training set contains 37,854 drone-view images, while the augmented dataset contains a total of 378,540 drone-view images. Furthermore, the satellite-view images are not augmented, as usually do not encounter the same weather-related distortions as drone-view images. Advanced image processing techniques are employed to simulate the selected weather conditions. For example, we simulate dark condition by adjusting brightness and contrast, overlay realistic raindrops and snowflakes using a particle system. University1652-plus ensures that each drone-view image in the training set is represented under ten different conditions, i.e., original and nine weather conditions, not only improving the model’s resilience to weather-related challenges but also better meeting the data requirements of the attention-based model.

### 3.3. Backbone Network: DINOv2

DINOv2 is a self-supervised learning method based on the Transformer architecture, designed to extract high-quality feature representations from images. The model employs a self-distillation strategy, where the student and teacher networks learn from each other, effectively capturing both global and local features from images without the use of labels. In this study, DINOv2 is used as the first-stage feature extractor. The input image $I\in R^{H\times W\times 3}$ is first divided into non-overlapping patches, which are mapped to the initial feature space $\;F_{0}\;$ (1) through the Patch Embed layer.

(1) $$F_{0}=\mathrm{PatchEmbedding}\left(I\right)$$

These features are then processed through Vision Transformer (ViT) blocks, where self-attention mechanisms compute the relationships between the patches. The self-attention calculation is given by Equation (2).

(2) $$A=\mathrm{softmax}\left(\frac{QK^{T}}{\sqrt{d_{k}}}\right)V$$

where $Q={\mathrm{Linear}}_{Q}\left(F\right)$, $K={\mathrm{Linear}}_{K}\left(F\right)$, and $V={\mathrm{Linear}}_{V}\left(F\right)$ are the query, key, and value matrices derived from the input feature F.

Within each ViT block, the features undergo non-linear transformations via an Multilayer Perceptron (MLP). This MLP consists of two fully connected layers, followed by the SwiGLU activation function. The mathematical representation of the MLP is as Equations (3) and (4).

(3) $$h_{1}=\mathrm{GELU}\left(W_{1}F+b_{1}\right)$$

(4) $$h_{2}=W_{2}h_{1}+b_{2}$$

where $W_{1}$ and $b_{1}$ are the weights and biases of the first layer, and $W_{2}$ and $b_{2}$ are the weights and biases of the second layer. The SwiGLU activation function is defined as Equation (5).

(5) SwiGLU(x)=ReLU(x)⊙Sigmoid(x)

To enhance robustness and generalization, DINOv2 adopts the masking strategy from iBOT, where random patches of the input image are masked for the student network while the teacher network receives the complete image. This strategy allows the student network to learn from incomplete information and improves its ability to generalize. The feature diversity is further enhanced by the use of the Sinkhorn–Knopp algorithm [57] to center the teacher network’s output.

Leveraging the powerful unsupervised learning capabilities of DINOv2, our AGEN can effectively capture deep image features and provide high-quality input for subsequent tasks.

### 3.4. LPN and Multi-Branch Classification

The extracted features after the image encoder are further processed by LPN in the backbone network with a multi-branch classification structure to gather environmental information. The use of LPN allows the model to extract more local feature details. These high-precision, fine-grained features are crucial for accurately interpreting and classifying complex scenes. Specifically, LPN is applied in the last layer of the backbone network using a specific feature pooling method. We first divide the input feature map into chunks to ensure each chunk captures the local details of the image. Then, adaptive average pooling or maximum pooling is used to extract key information from each chunk. To avoid overlapping and redundancy, the pooling process is adjusted according to the size and location of the chunk. Formally, given an input feature map $F\in R^{C\times H\times W}$, it is partitioned into *k* non-overlapping regions ${\left\{F_{i}\right\}}_{i=1}^{k}$, and the pooled feature for each region is obtained as Equation (6).

(6) $$f_{i}=\mathrm{Pool}\left(F_{i}\right),\;f_{i}\in R^{C}$$

In order to preserve spatial detail and structural semantics, these local features are concatenated into a unified representation as Equation (7).

(7) $$f=\mathrm{Concat}(f_{1},f_{2},\ldots ,f_{k})$$

Finally, all pooled feature chunks are stitched together to form a feature map with rich local details. The multi-branch classification header responsible for categorizing these fine-grained features consists of multiple branches, each corresponding to a part of the feature map. Each branch first performs a spreading operation on its corresponding feature block, which is followed by using a separate classifier. Mathematically, each branch applies an independent classifier $\phi_{i}\left(\cdot \right)$ to its input $f_{i}$, producing a prediction as in Equation (8).

(8) $${\hat{y}}_{i}=\phi_{i}\left(f_{i}\right)$$

**Figure 4:**
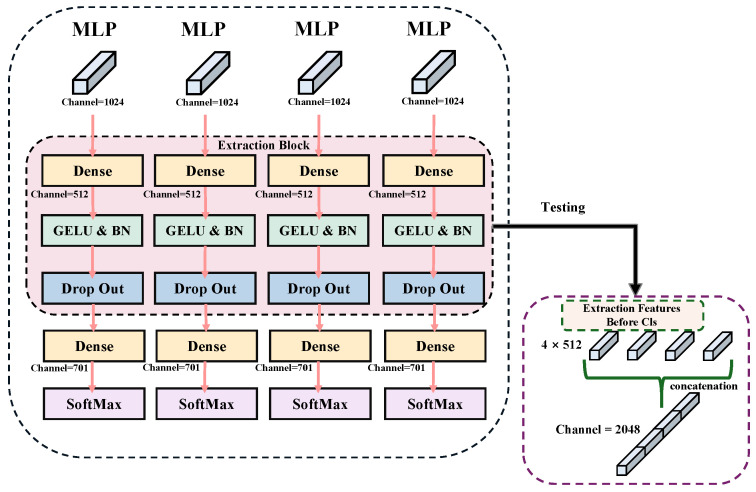
Multiple classifiers branch.

During training, the classification loss from each branch is directly summed to form the final LPN loss as Equation (9), allowing the model to fully leverage the contribution of each local feature region.

(9) $$L_{\mathrm{LPN}}=\sum_{i=1}^{k}\mathrm{CE}\left({\hat{y}}_{i},y\right)$$

where *y* is the ground-truth label. This allows each branch’s classifier to focus on specific local features, improving classification accuracy and fine-grained feature interpretation. During training, classification results from each branch are integrated to optimize overall model performance. During inference, classification results from each branch are stacked to provide detailed outputs, as illustrated in Figure 4.

This structure fully leverages the advantages of the LPN and the multi-branch classification head, addressing the limitations of traditional feature extraction methods in capturing fine-grained features in complex scenes. As a result, the model is able to more accurately perform target localization and recognition under adverse conditions, enhancing classification accuracy and robustness.

### 3.5. Fuzzy PID Control Mechanism

The fuzzy proportional–integral–derivative (PID) controller integrates the strengths of classical PID control and fuzzy logic systems, making it particularly suitable for handling complex systems characterized by nonlinearity, time variance, and uncertainty. In contrast to traditional optimization strategies that depend on fixed hyperparameters, the fuzzy PID controller adaptively determines the proportional (P), integral (I), and derivative (D) gains by evaluating the current error, its temporal variation, and the accumulated error over time. This enables more robust and adaptive control behavior. In recent years, as deep learning models have become increasingly complex, the training process has exhibited strong non-linear and dynamic properties, such as slow gradient updates in early stages and oscillations in later stages. Conventional learning rate scheduling strategies (e.g., preset decay or cosine annealing) often fail to simultaneously ensure both fast convergence and stable training. To address these challenges, this study introduces a fuzzy PID control mechanism into the deep learning optimization process to enhance the model’s adaptive learning capacity.

The optimization strategy proposed in this study introduces a fuzzy PID controller to dynamically adjust the relative importance of each training epoch in the global loss computation. Specifically, let $L_{t}$ denote the average loss of the *t*-th epoch. The input error signal to the controller is defined as a weighted combination of recent epoch losses as Equation (10).

(10) $$e\left(t\right)=a\cdot L_{t}+b\cdot L_{t-1}+c\cdot L_{t-2},\;\mathrm{where}\;a+b+c=1,\;a>b>c$$

The fuzzy PID controller computes a control signal based on this error, its cumulative history, and its temporal variation as Equation (11).

(11) $$u\left(t\right)=S_{P}\left(t\right)\cdot e\left(t\right)+S_{I}\left(t\right)\cdot \sum_{k=0}^{t}e\left(k\right)+S_{D}\left(t\right)\cdot \left(e(t)-e(t-1)\right)$$

The output control signal u(t) (12) is then normalized using a softmax function to yield the weight assigned to epoch *t*:

(12) $$w\left(t\right)=\frac{\exp(u(t))}{\sum_{\tau =t-m}^{t}\exp\left(u\left(\tau \right)\right)}$$

where *m* denotes the number of historical epochs considered in the weighting window. The global loss (13) is reformulated as a weighted sum over multiple recent epochs:

(13) $$L_{\mathrm{global}}=\sum_{\tau =t-m}^{t}w\left(\tau \right)\cdot L_{\tau }$$

This weighting strategy allows the controller to distinguish and emphasize training stages with slower convergence or larger errors. When an epoch exhibits higher average loss, its corresponding control signal u(t) increases, leading to a higher normalized weight w(t). As a result, the training process is guided to focus more on suboptimal epochs that require greater learning attention.

From an optimization perspective, assuming a learning rate η, the parameter update rule can be approximated as Equation (14).

(14) $$\theta_{t+1}=\theta_{t}-\eta \cdot \sum_{\tau =t-m}^{t}w\left(\tau \right)\cdot \nabla_{\theta }L_{\tau }$$

Compared with uniform weighting (e.g., w(τ)=1/(m+1)), the fuzzy PID-based weighting mechanism increases the influence of more error-prone epochs on the update direction. This yields a more effective descent in the expected loss gradient (15).

(15) $$\nabla L_{\mathrm{global}}{\left(\theta_{t}\right)}^{⊤}\cdot \sum_{\tau }w\left(\tau \right)\cdot \nabla_{\theta }L_{\tau }>\nabla L_{\mathrm{global}}{\left(\theta_{t}\right)}^{⊤}\cdot \frac{1}{m+1}\sum_{\tau }\nabla_{\theta }L_{\tau }$$

Therefore, the proposed epoch-level weighting approach, guided by fuzzy PID control, enhances convergence speed in the early training phase by emphasizing high-error epochs while maintaining smooth and stable optimization behavior in later stages. This mechanism effectively restructures the optimization trajectory based on training dynamics, promoting more efficient and robust learning.

### 3.6. Loss Function Optimization

In extreme weather conditions such as heavy rain and dense fog, significant discrepancies between drone-view and satellite-view images introduce substantial nonlinearity, time variance, and uncertainty into the training process. As the dataset is expanded, the number of samples per epoch becomes limited relative to the overall dataset size, resulting in each epoch covering only a small fraction of the entire data distribution. Consequently, it becomes challenging for a single epoch to fully capture the variations across geographic regions, meteorological conditions, and perspective differences. This problem is particularly pronounced under extreme weather scenarios, substantially impairing convergence speed and training stability.

To address these challenges, an Adaptive Error Control (AEC) module is introduced to improve the effectiveness of the conventional cross-entropy loss function during training. By incorporating a fuzzy PID control mechanism, the AEC module adaptively adjusts loss weights through proportional (P), integral (I), and derivative (D) control strategies. The P control enables real-time adjustment according to the current error, enhancing sensitivity to abrupt variations. The I control accumulates historical error information to correct long-term biases, while the D control captures the trend of error evolution to suppress fluctuations caused by environmental perturbations. By jointly considering multiple epochs, the AEC mechanism effectively expands the model’s temporal perception, allowing it to better integrate historical information and improve robustness under extreme conditions.

**Figure 5:**
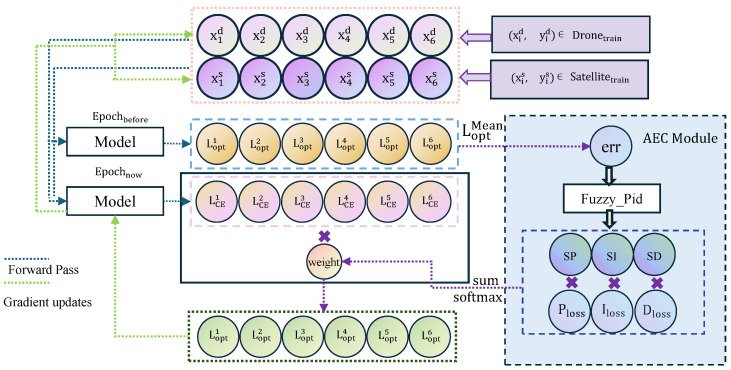
Loss function optimization.

The overall training process, as illustrated in Figure 5, demonstrates how historical information and the Adaptive Error Control (AEC) mechanism optimize parameters using six data samples. The dataset consists of drone-view and satellite-view image samples, denoted as $\left(x_{i}^{d},y_{i}^{d}\right)$ and $\left(x_{i}^{s},y_{i}^{s}\right)$, respectively, to address multi-view image-matching tasks. After each sample undergoes forward propagation, the cross-entropy loss function $L_{\mathrm{CE}}$ is used to calculate the error between the model’s predictions and the ground truth labels, defined as Equation (16).

(16) $$L_{\mathrm{CE}}=-\sum_{i=1}^{N}y_{i}\log\left({\hat{y}}_{i}\right)$$

where $y_{i}$ denotes the ground-truth label and ${\hat{y}}_{i}$ denotes the predicted probability.

To enhance adaptability during training, control parameters SP, SI, and SD are dynamically generated based on fuzzy logic reasoning. These parameters adjust the loss weights in subsequent iterations according to the magnitude and dynamics of the current loss. Samples with higher losses are assigned greater weights, directing the optimization to focus on more difficult learning instances.

The AEC module dynamically computes the control signal *u* via the fuzzy PID controller, as formulated in Equation (17):

(17) $$u=\mathrm{SP}\times \mathrm{err}+\mathrm{SI}\times I_{\mathrm{loss}}+\mathrm{SD}\times D_{\mathrm{loss}}$$

where SP, SI, and SD correspond to the proportional, integral, and derivative components, respectively. These components reflect the dynamic adjustment of the control signal according to both the current and historical training states. Their values are not statically assigned but are dynamically adjusted during training through a fuzzy logic controller. This adaptive mechanism aims to minimize the discrepancy between predictions and ground-truth labels by responding to variations in the loss trend, thereby enhancing the model’s ability to adapt to changes in training dynamics. Moreover, the integral term $I_{\mathrm{loss}}$ aggregates recent epoch losses to capture long-term trends and correct potential bias, as formulated in Equation (18).

(18) $$I_{\mathrm{loss}}=\sum_{j=1}^{T}\lambda_{j}\cdot {\mathrm{loss}}_{t-j}$$

where $\lambda_{j}$ are decay coefficients (with $\sum_{j}\lambda_{j}=1$), and T≤3 represents the number of past epochs considered. This accumulation enhances robustness to transient fluctuations by preserving temporal context. The derivative term $D_{\mathrm{loss}}$ is computed as the difference between the most recent two epoch losses in Equation (19).

(19) $$D_{\mathrm{loss}}=\mathrm{last}\_\mathrm{loss}-\mathrm{last}\_\mathrm{last}\_\mathrm{loss}$$

This term evaluates the velocity of loss change and contributes to damping oscillations, thereby stabilizing the optimization trajectory under extreme weather disturbances.

The current error err is dynamically computed based on the losses of recent epochs, following the conditions outlined below:

- When epoch=warm_epoch+1, only the previous epoch’s loss is available: err=last_loss.
- When epoch=warm_epoch+2, the error is a weighted combination of the previous two epochs: err=a·last_loss+(1−a)·last_last_loss.
- When epoch>warm_epoch+2, the error incorporates the last three epochs: err=a·last_loss+b·last_last_loss+c·last_last_last_loss with a+b+c=1.

(20) $$\mathrm{err}=\left\{\begin{array}{c} \mathrm{last}\_\mathrm{loss},\\ a\cdot \mathrm{last}\_\mathrm{loss}+(1-a)\cdot \mathrm{last}\_\mathrm{last}\_\mathrm{loss},\\ a\cdot \mathrm{last}\_\mathrm{loss}+b\cdot \mathrm{last}\_\mathrm{last}\_\mathrm{loss}+c\cdot \mathrm{last}\_\mathrm{last}\_\mathrm{last}\_\mathrm{loss}. \end{array}\right.$$

This formulation ensures a balanced incorporation of historical information, enabling the model to maintain sensitivity to both short-term and long-term variations during training.

The weights for each sample are obtained by applying a softmax normalization to the control signal *u*, as shown in Equation (21):

(21) weights=softmax(u)

The optimized per-sample loss $L_{\mathrm{opt}}^{i}$ is then computed by weighting the cross-entropy loss for each sample as shown in Equation (22):

(22) $$L_{\mathrm{opt}}^{i}={\mathrm{weights}}_{i}\cdot L_{\mathrm{CE}}^{i},\;i=1,2,\ldots ,n$$

where *n* denotes the batch size, $L_{\mathrm{CE}}^{i}$ is the original cross-entropy loss, and ${\mathrm{weights}}_{i}$ is the corresponding adaptive weight for sample *i*. The weighted losses are then used for gradient updates on a per-sample basis, ensuring that more challenging samples receive greater attention during optimization.

By integrating historical losses across multiple epochs and dynamically adjusting the optimization trajectory, the proposed AEC module significantly improves convergence efficiency, enhances model stability, and strengthens generalization under extreme weather and complex viewpoint variations.

## 4. Experiments

### 4.1. Datasets Description

University-1652 [26] includes 1652 locations across 72 universities worldwide, offering a diverse range of urban and campus environments. This multi-view, multi-source methodology allows for a robust examination of geo-localization from diverse perspectives, presenting unique challenges. To support this framework, satellite-view images are acquired by projecting geographic coordinates from Wikipedia metadata of university buildings onto Google Maps, with one high-resolution image per building serving as a static reference. Drone-view images are generated via simulated flights around 3D building models from Google Earth, where a virtual drone follows a three-orbit spiral trajectory descending from 256 m to 121.5 m. Video is recorded at 30 fps, and one frame is extracted every 15 frames, yielding 54 images per building that capture diverse viewpoints and realistic scales. This approach captures diverse viewpoints and scales, thereby providing high-quality and realistic samples for cross-view matching tasks.

**Figure A1:**
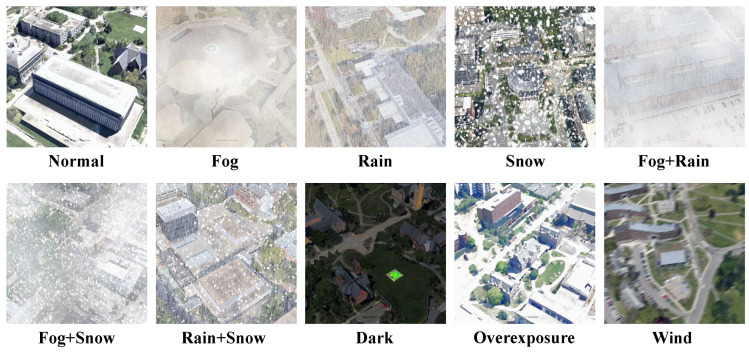
Multiple weather samples in University160k-WX, with diverse building types.

University160k-WX [13] dataset is presented by the ACM MM2024 Workshop on UAVs in Multimedia, aiming to simulate real-world geo-localization scenarios by introducing multi-weather cross-view variants. University160k-WX extends the University-1652 dataset by adding 167,486 satellite-view gallery distractors and includes weather variations such as fog, rain, snow, and multiple weather compositions, as shown in Figure A1. This significantly increases the complexity and challenge for representation learning.

University1652-Plus, an extensive expansion of the University-1652 dataset, introduces nine extreme weather conditions (darkness, rain, fog, snow, fog and rain, fog and snow, sleet, light, and wind) to enhance model robustness for cross-view geo-localization. This augmented dataset expands the original 37,854 drone-view images to 378,540 images, simulating challenging environments. We generate fog by combining Gaussian blur and particle effects and overlay rain and snow effects when applicable. Simulate different light intensities and direction changes, and apply motion blur to simulate wind effects.

SUES-200 [27] is a cross-view image-matching dataset that contains 24,120 images captured by UAVs at four different altitudes and corresponding satellite views of the same target scene. All images are acquired in realistic environments with multiple types of scenes, including real-world lighting, shadow transformations, and interference. In order to address the shortcomings of existing public datasets, SUES-200 also takes into account the differences that occur when UAVs shoot at different altitudes. More over, the SUES-200 dataset contains 200 distinct locations, each associated with one satellite-view image and 50 drone-view images. Satellite-view images are sourced from AutoNavi Map and Bing Maps, with a single high-resolution image 512×512 assigned per location. Drone-view images are captured along a predefined curved trajectory with varying altitudes to ensure multi-angle coverage. Each flight video yields 50 uniformly sampled frames, and the original resolution of 1080×1080 is preserved to retain visual detail and minimize information loss. The details of the datasets used in this paper are provided in Table 1, Table 2 and Table 3, where “Locations” denotes the number of distinct geographic spots within the dataset.

### 4.2. Implementation Details

In this investigation, the network initialization is based on DINOv2’s large version, with weights pre-trained on the ImageNet dataset, serving as a cornerstone for the transfer learning paradigm. Each input image is first resized to 518×518 pixels using bicubic interpolation to ensure consistency in spatial resolution. To enhance generalization under diverse imaging conditions, the pipeline applies 10-pixel edge padding to suppress boundary artifacts, performs random cropping to introduce spatial variation, and uses horizontal flipping to increase viewpoint robustness. For satellite-view images, random affine transformations with up to ±90^∘^ rotation simulate sensor-induced misalignment and orbital drift. Image normalization with ImageNet statistics stabilizes training dynamics. The network is developed using the PyTorch framework (version 2.5.0, CUDA 11.8) and optimized with the Rectified Adam (RAdam) optimizer. This optimizer uses a weight decay parameter set to $1\times 10^{-4}$. The initial learning rate is set to $1\times 10^{-4}$ for the backbone layer and $1\times 10^{-4}$ for the classification layer. Computational experiments are conducted on an NVIDIA A40 GPU with 48 GB of memory.

### 4.3. Comparison with Other Data Augmentation Methods

The original University-1652 training set contained 37,854 drone viewpoint images. A total of 378,540 drone view images were generated from this data expansion. The satellite view images were not augmented because they are not subject to the same weather-related effects as the drone view images. To simulate these selected weather conditions, advanced image processing techniques are used in this paper. Three major image processing libraries, the imgaug, OpenCV, and Pillow, are used to compare the matching results.

In this paper, a series of enhancement operations using imgaug library are defined to simulate dark environments by adjusting brightness and contrast, superimposing real raindrops and snowflakes using a particle system, generating fog by combining Gaussian blur and particle effects, and superimposing rain and snow effects when appropriate. In addition, different light intensities and direction changes are simulated, and wind effects are simulated by motion blur to enhance the realism of the image.

OpenCV is mainly used to implement a variety of weather effects. In this paper, a black rain layer of the same size as the original image is created to simulate rainfall by randomly generating a certain number of white lines. An all-white fog layer is created and combined with the original image using a weighted average method; the blending ratio is adjusted to simulate different intensities of mist and haze; white circles are drawn randomly on an empty white snow layer to form snowflakes and combined with the original image for superimposition; bright light and darkness are simulated by adjusting the brightness and contrast of the image; and affine transforms are combined with image translation to simulate the effect of wind.

The Pillow library (version 10.2.0), on the other hand, provides easy-to-use image processing functions. Rain and snow are simulated by drawing white lines and circles at random locations. For fog simulation, Pillow generates a mist effect by weighted blending an all-white layer with the original image. The contents of the original image are duplicated, and then Gaussian blur is used to generate the visual effect of wind. In addition, Pillow is used for simple brightness and contrast adjustments to further enhance the image’s diversity.

The effectiveness of data augmentation methods was evaluated based on the Recall@1, Recall@5, and Recall@10 accuracy scores. As shown in Table 4, the imgaug library achieved Recall@1, Recall@5, and Recall@10 scores of 85.58%, 92.83%, and 94.02%. In comparison, using OpenCV and Pillow resulted in lower scores. Specifically, imgaug improved Recall@1 by 10.52% and 4.83%, Recall@5 by 8.30% and 4.28%, and Recall@10 by 7.24% and 3.10% over OpenCV and Pillow, respectively. These results indicate that the weather augmentation method using imgaug significantly enhances model performance. Therefore, we choose the imgaug library as the preferred library due to its powerful customization capabilities and rich enhancement features. This may provide useful guidance for future deep-learning applications under complex weather conditions.

To validate the effectiveness of the data augmentation method, we conducted experiments using the Swin Transformer model [38]. First, we applied a multi-weather denoising method [19] to process the weather-noisy images in the University160K-WX training set, and then we trained the model on the denoised training set before testing. In contrast, the second experiment involved training directly on the original training set, which contains weather noise, in order to adapt the model to extreme weather conditions. The experimental results, as shown in Table 5, indicate that when training directly on the weather-noisy training set, the model’s performance on the test set achieved Recall@1 of 62.71%, Recall@5 of 74.07%, and Recall@10 of 77.24%. On the other hand, when training on the denoised training set, the model achieved Recall@1 of 53.48%, Recall@5 of 64.68%, and Recall@10 of 68.14%. These results further confirm that while image denoising can effectively remove weather noise, it may also lead to the loss of certain image information, which impacts the matching performance.

### 4.4. Comparison with Other Methods

In the context of our study, the drone view target localization task refers to the process of matching a target in a satellite view with the corresponding position in a drone view. On the other hand, drone navigation applications involve the reciprocal task of matching the drone view with the corresponding location in the satellite view.

As shown in Table 6, the comparative efficacy of the proposed AGEN network is first evaluated using the University-1652 dataset. Notably, when applied to the task of drone-view target localization, the AGEN model achieves a Recall@1 accuracy of 95.43% and an AP of 96.18%. Furthermore, for the drone navigation application task, the network achieves a Recall@1 accuracy of 96.72% and an AP of 95.52%. The performance has surpassed the state-of-the-art methods, e.g., WELN and SRLN. Specifically, WELN achieved a Recall@1 accuracy of 92.87% and an AP of 94.00% for drone-view target localization task and 93.46% Recall@1 with an AP of 93.25% for the drone navigation application task. Similarly, SRLN reached a Recall@1 accuracy of 92.70% and an AP of 93.77% for the drone-view target localization task, while in the drone navigation application task, it achieved 95.14% Recall@1 and an AP of 91.97%. Overall, these results demonstrate an improvement in performance, highlighting the effectiveness of our network.

We further perform a systematic comparison of AGEN with existing algorithms under the SUES-200 dataset. The result is illustrated in Table 7. For the drone viewpoint target localization task, the AGEN network shows a significant improvement in Recall@1 accuracy, which is 94.38% at 150 m above sea level and reaches a maximum point of 97.12% at 300 m above sea level. At the same time, the AP score gradually increases from 95.58% to 97.81%. Compared with the state-of-the-art model CCR [64], the accuracy of Recall@1 increased by 7.30% and the AP score by 6.03% at 150 m above sea level, while at 300 m above sea level, the accuracy of Recall@1 increased by 0.30% and the AP score increased by 0.42%. Similarly, in the drone navigation task example, the AGEN network achieves Recall@1 accuracy of 97.50% at 150 m and 96.52% at 300 m above sea level. Meanwhile, the AP score increased from 92.58% to 95.36%. These statistics not only outperform the Safe-Net [63] model, which has AP scores between 86.36% and 95.67%, but also emphasize the excellent uniform performance of the AGEN network at all flight altitudes. The insights gained from these data demonstrate the leading position of the AGEN model in terms of accuracy and stability, thus significantly advancing the field of geographic information.

To validate the effectiveness of the proposed Adaptive Error Control (AEC) module, we conduct comprehensive comparative experiments on the University-1652 dataset. Three representative backbone networks are selected for evaluation, including the conventional convolutional architectures VGG16 and ResNet34/50, as well as the Vision Transformer (ViT), pre-trained with enhanced regularization. As shown in Table 8, the integration of the AEC module consistently improves the performance across all models in both the drone viewpoint target localization task and the drone navigation task, as measured by Recall@1, Recall@5, Recall@10, and AP. In the table, a check mark (✓) indicates that the AEC module is applied. For instance, under the ViT architecture, AEC improves the Recall@1 from 83.72% to 85.68% and the AP from 86.08% to 87.76% in the drone viewpoint target localization task while yielding a 2.0% improvement in Recall@1 in the drone navigation task. Similarly, under the ResNet50 architecture, the Recall@1 increases from 65.62% to 68.87% and AP from 69.75% to 72.80% in the localization task; in the navigation task, Recall@1 improves from 77.46% to 82.17%, and AP from 65.15% to 69.77%. These results indicate that the AEC module promotes more effective learning of task-relevant knowledge, contributing to improved feature representations and overall matching performance.

Since University160k-WX only provides a mask-processed test set, our model uses University1652-Plus, an extension of the University-1652 training set, during the debugging phase. As shown in Table 9, we submit our best Recall@1 results for the challenge using the University160k-WX test set, achieving a Recall@1 accuracy of 91.71%, Recall@5 accuracy of 96.36%, and Recall@10 accuracy of 97.03%. Compared with the WELN model, our approach improved Recall@1 by 6.04%, Recall@5 by 3.55%, and Recall@10 by 2.99%. In addition, compared with the EDTA model, which achieved 85.08% at Recall@1, 91.18% at Recall@5, and 92.72% at Recall@10, our method shows a substantial performance gain of 6.63%, 5.18%, and 4.31%, respectively. These results clearly demonstrate the superior generalization ability and robustness of our proposed method under diverse and challenging weather conditions.

### 4.5. Ablation Study

In our ablation study, as shown in Table 10, we evaluate the impact of various components, including shared weights, LPN, training on University1652-Plus, and the AEC module, with performance results on both the University160k-WX and University-1652 datasets.

The baseline model is defined as the DINOv2-large version without shared weights, trained solely on the University-1652 dataset. This initial model achieves a Recall@1 of 30.67% on University160k-WX and 91.71% on University-1652, providing a reference point for measuring the effectiveness of added components. Adding shared weights significantly boosts performance, raising Recall@1 to 46.09% on University160k-WX and to 93.98% on University-1652, indicating the benefit of shared feature learning across views. Incorporating LPN further improves results, particularly on University160k-WX, where Recall@1 increases to 55.07%, highlighting LPN’s ability to capture fine-grained details in diverse conditions. Training on the University1652-Plus dataset yields the most substantial improvement, with Recall@1 reaching 88.80% on University160k-WX and 94.65% on University-1652, demonstrating the advantage of data expansion in model robustness. Finally, integrating the AEC module leads to further gains, achieving Recall@1 of 91.71% on University160k-WX and 95.43% on University-1652, underscoring the module’s impact on enhancing adaptability and accuracy under challenging conditions.

These findings highlight the contribution of each component to improving model robustness and accuracy across diverse datasets and weather conditions.

### 4.6. Visualization

**Figure 6:**
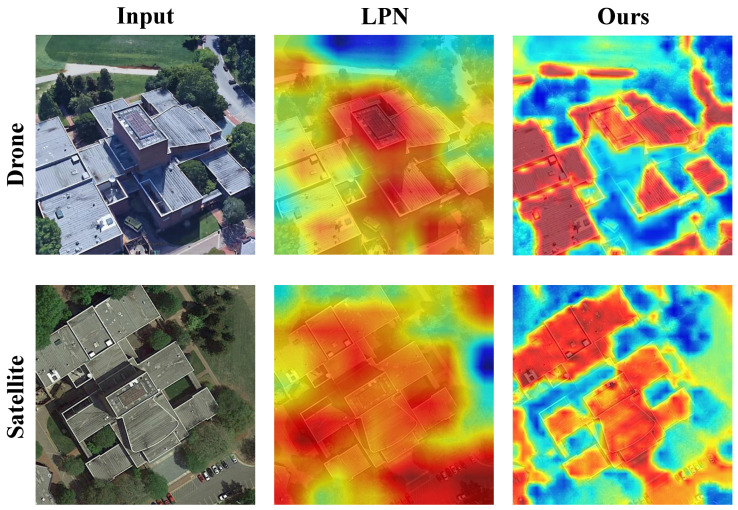
Heatmap comparison between LPN and our method. In the heatmap, red, yellow, and blue correspond to high, moderate, and low attention intensities, respectively. Darker shades within the same color indicate stronger attention.

In order to better demonstrate the matching effect of AGEN, we plot the heat maps of the LPN methods and the proposed AGEN, as shown in Figure 6. The images in the first column are the input images from drone view and satellite view. The images in the second column are the heatmaps of LPN [25]. The images in the last column are our heatmaps. The presented comparison illustrates the effectiveness of our proposed method in enhancing image matching between drone and satellite views. The input images clearly show detailed architectural features, while the corresponding heatmaps reveal significant differences in model performance. The previous method’s heatmaps, although providing some activation, lack precision in capturing critical details, especially around the edges of the buildings. In contrast, our method produces heatmaps that better highlight the intricate structures and contours of the buildings, showcasing improved clarity and accuracy.

**Figure 7:**
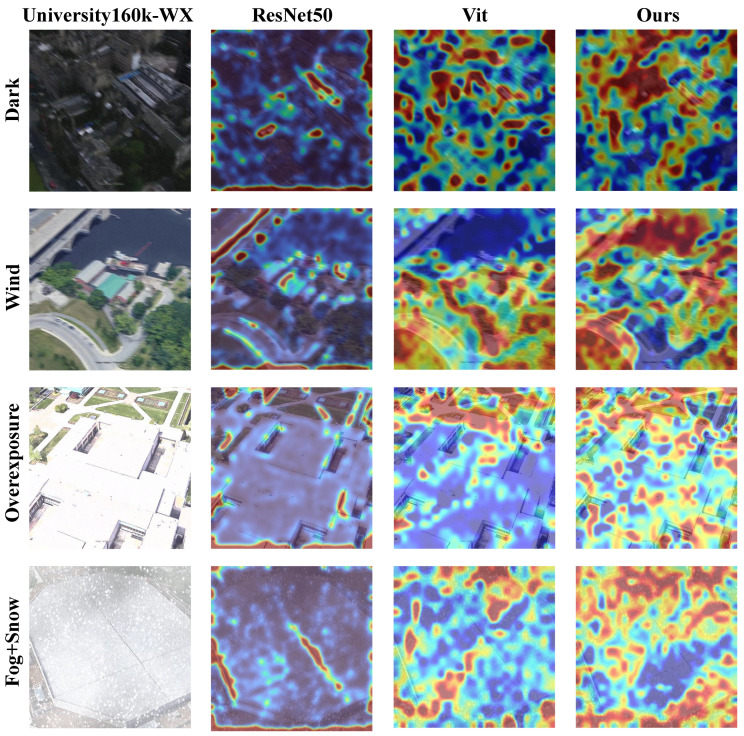
Heatmap comparison of different backbone networks under extreme weather conditions. In the heatmap, red, yellow, and blue correspond to high, moderate, and low attention intensities, respectively. Darker shades within the same color indicate stronger attention.

To further investigate the weather robustness of different backbone architectures, we visualize and compare the attention heatmaps generated by ResNet50 [68], ViT [69], and our proposed DINOv2-based model under four representative extreme weather conditions from the University160k-WX dataset. These conditions include wind, overexposure, fog and snow, and darkness. This comparison allows us to assess how each model attends to salient regions under different types of environmental degradation. As illustrated in Figure 7, the ResNet50 model tends to produce scattered and low-contrast attention regions, especially in low-visibility scenes. The ViT backbone exhibits moderately improved attention localization but remains sensitive to adverse illumination and occlusion. In contrast, the DINOv2-based model consistently captures focused, object-aligned attention maps across all conditions, demonstrating its superior capacity for learning weather-invariant representations. This qualitative comparison complements the quantitative results and further supports the effectiveness of our approach in challenging environments.

## 5. Discussion

Although the proposed method performs reliably across a range of extreme weather scenarios, it still presents several limitations that merit further investigation.

First, although the proposed method shows strong robustness under extreme weather, the test set uses a masked evaluation protocol without revealing specific weather labels, limiting fine-grained analysis under conditions like fog or rain. Future work could introduce datasets with explicit annotations to support targeted evaluation and model improvement.

Second, the dataset assumes that nine representative weather types sufficiently capture major visual degradations and use synthetic augmentation to simulate extreme conditions. However, a potential domain gap may exist between simulated and real-world data, which can hinder generalization. To address this, future research may define a more granular taxonomy of weather types or collect and label real remote sensing imagery under adverse conditions, thereby enhancing model reliability and deployment robustness.

Finally, despite covering typical extreme conditions, the current weather types are limited. In more complex scenarios, performance may degrade. Exploring generative models such as the diffusion model [74] could help synthesize realistic weather data and facilitate the restoration of clean images from weather-degraded inputs. These capabilities support the creation of high-fidelity datasets and enhance both the generalization and interpretability of geo-localization models in real-world applications.

## 6. Conclusions

In this paper, we propose a cross-view geo-localization method, AGEN, for robust matching performance under extreme weather conditions. The AGEN framework designs a DINOv2-based backbone for robust feature extraction via contrastive learning. We further integrate the LPN module to enhance precision in recognizing fine-grained geographical features. To enhance the robustness of AGEN under extreme weather scenarios, we designed the AEC module, which dynamically adjusts the loss weights to improve model adaptability in challenging conditions. Additionally, we extend the University-1652 dataset to include various weather conditions, creating a more comprehensive testing environment. Experimental results on the original University-1652, University160k-WX, and SUES-200 datasets validate the effectiveness of our method. Notably, AGEN achieves a Recall@1 of 95.43% on University-1652 for the drone-view target localization task and 91.71% on University160k-WX under extreme weather conditions, demonstrating its robustness and superior retrieval capability across diverse scenarios. We hope our exploration, such as the AEC module, will inspire further research, driving more resilient and practical applications under real-world adverse weather conditions.

## Figures and Tables

**Table 1:** Training set and testing set of the University-1652 dataset.

Views	Locations	Images
	**Training Set**	
Drone	701	37,854
Satellite	701	701
	**Testing Set**	
Drone Query	701	37,854
Satellite Query	701	701
Drone Gallery	951	51,355
Satellite Gallery	951	951

**Table 2:** Training dataset on University1652-Plus and testing dataset on name-masked University160k-WX.

Views	Locations	Images
	**Training Dataset**	
Drone	701	378,540
Satellite	701	701
**Views**	**Images**	
	**Testing Dataset**	
Drone Query	37,855	
Satellite Gallery	168,437	

**Table 3:** Training set and testing set of the SUES-200 dataset.

Views	Locations	Images at Each Height	Total
**Training Set**			
Drone	120	6000	24,000
Satellite	120	-	120
**Testing Set**			
Drone Query	80	4000	16,000
Satellite Query	80	-	80
Drone Gallery	200	10,000	40,000
Satellite Gallery	200	-	200

**Table 4:** Comparison of the effectiveness of data augmentation methods. The best results are in bold.

Method	Recall@1	Recall@5	Recall@10
OpenCV	75.06	84.53	86.78
Pillow	80.75	88.55	90.92
**Imgaug**	**85.58**	**92.83**	**94.02**

**Table 5:** Performance Comparison of swin transformer with and without weather noise removal on the University160K-WX dataset. The best results are in bold. A checkmark (✓) indicates that the weather noise removal method is applied.

Backbone	Weather Noise Removal	Recall@1	Recall@5	Recall@10
Swin Transformer [38]	✓	53.48	64.68	68.14
**Swin Transformer**		**62.71**	**74.07**	**77.24**

**Table 6:** Comparison with the state-of-the-art methods on the University-1652 dataset. The best results are in bold.

Method	Drone → Satellite		Satellite → Drone	
	Recall@1	AP	Recall@1	AP
LPN [25]	75.93	79.14	86.45	74.79
FSRA (k = 1) [29]	82.25	84.82	87.87	81.53
FSRA (k = 3) [29]	84.51	86.71	88.45	83.37
PAAN [58]	84.51	86.78	91.01	82.28
Swin-B + DWDR [59]	86.41	88.41	91.30	86.02
MBF [60]	89.05	90.61	93.15	88.17
GeoFormer [61]	89.08	90.83	92.30	88.54
MCCG [62]	89.64	91.32	94.30	89.39
Safe-Net [63]	86.98	88.85	91.22	86.06
CCR [64]	92.54	93.78	95.15	91.80
SRLN [34]	92.70	93.77	95.14	91.97
WELN [65]	92.87	94.00	93.46	93.25
**AGEN**	**95.43**	**96.18**	**96.72**	**95.52**

**Table 7:** Comparison with state-of-the-art results on SUES-200. The best results are in bold.

Drone → Satellite								
Method	150 m		200 m		250 m		300 m	
	Recall@1	AP	Recall@1	AP	Recall@1	AP	Recall@1	AP
SUES-200 Baseline [27]	55.65	61.92	66.78	71.55	72.00	76.43	74.05	78.26
LCM [66]	43.42	49.65	49.42	55.91	54.47	60.31	60.43	65.78
LPN [25]	61.58	67.23	70.85	75.96	80.38	83.80	81.47	84.53
FSRA [29]	68.25	73.45	83.00	85.99	90.68	92.27	91.95	93.46
Safe-Net [63]	81.05	84.76	91.10	93.04	94.52	95.74	91.95	93.46
MCCG [62]	82.22	85.47	89.38	91.41	93.82	95.04	94.57	95.60
CCR [64]	87.08	89.55	**93.57**	**94.90**	95.42	96.28	96.82	97.39
**AGEN**	**94.38**	**95.58**	91.78	93.35	**95.75**	**96.65**	**97.12**	**97.81**
SUES-200 Baseline [27]	75.00	55.46	85.00	66.05	86.25	69.94	88.75	74.46
LCM [66]	57.50	38.11	68.75	49.19	70.50	49.94	75.00	59.36
LPN [25]	83.75	66.78	88.75	75.01	92.50	81.34	92.50	85.72
FSRA [29]	83.75	76.67	90.00	85.34	93.75	90.17	95.00	92.03
CCR [64]	92.50	88.54	97.50	**95.22**	**97.50**	**97.10**	97.50	**97.49**
MCCG [62]	93.75	89.72	93.75	92.21	96.25	96.14	**98.75**	96.64
Safe-Net [63]	97.50	86.36	96.25	92.61	**97.50**	94.98	**98.75**	95.67
**AGEN**	**97.50**	**92.58**	**97.50**	93.11	96.25	94.40	96.52	95.36

**Table 8:** Evaluation of the AEC module under different backbone architectures on the University-1652 dataset.

Backbone	Image Size	AEC	Drone → Satellite				Satellite → Drone			
			Recall@1	Recall@5	Recall@10	AP	Recall@1	Recall@5	Recall@10	AP
VGG16 [67]	224×224		28.05	44.75	53.02	32.30	37.52	43.22	46.65	25.66
VGG16	224×224	✓	29.39	46.77	55.01	33.72	37.66	43.65	47.65	26.09
ResNet34 [68]	224×224		50.69	71.71	78.99	55.56	66.62	75.46	77.89	51.82
ResNet34	224×224	✓	53.20	74.83	81.66	58.14	68.33	77.03	80.17	52.83
ResNet50 [68]	224×224		65.62	83.77	88.73	69.75	77.46	83.02	86.16	65.15
ResNet50	224×224	✓	68.87	86.40	90.48	72.80	82.17	86.45	88.16	69.77
ViT [69]	224×224		83.72	94.24	95.98	86.08	89.87	93.30	94.29	84.66
ViT	224×224	✓	85.68	95.00	96.51	87.76	91.87	94.72	95.44	85.63

**Table 9:** Comparison with other novel methods on University160K-WX. The best results are highlighted in bold.

Method	Backbone	Recall@1	Recall@5	Recall@10
LPN [25]	ResNet50 [68]	7.98	10.25	11.21
CAGM [70]	ConvNeXt [71]	78.56	87.14	89.08
MCGF [22]	EVA02 [41]	84.68	91.36	93.00
EDTA [72]	Swin ViT v2 [73]	85.08	91.18	92.72
WELN [65]	EVA02 [41]	85.58	92.83	94.02
**AGEN**	**DINOv2** [24]	**91.71**	**96.36**	**97.03**

**Table 10:** Ablation Experiments.

Method	University160K-WX		University-1652	
	Recall@1	Recall@5	Recall@1	Recall@5
Baseline	30.67	38.06	91.71	97.69
+Shared weights	46.09	54.74	93.98	98.13
+LPN	55.07	63.52	94.51	98.20
+University1652-Plus	88.80	94.74	94.65	98.74
+AEC	91.71	96.36	95.43	98.76

## Data Availability

The data presented in this study are available on request from the first author.

## Abbreviations

The following abbreviations are used in this manuscript:

LPN	Local Pattern Network
AEC	Adaptive Error Control
IMU	inertial measurement unit
DID-MDN	Density-Aware Single Image De-raining Using a Multi-stream Dense Network
SRLN	Scale-aware Radial Local Network
ViT	Vision Transformer
PID	Proportional–Integral–Derivative
P	Proportional
I	Integral
D	Derivative
EDTA	Evaluating Domain Translation Approaches for Drone-Based Geo-Localization in Adverse Weather
CAGM	A ConvNeXt-Based Network for Aerial-view Geo-localization in Multiple Environment

## Appendix A

The University160k-WX dataset is an extended version of the University-1652 dataset, incorporating nine representative extreme weather conditions, including fog, rain, snow, and their compound variants. The images span a wide range of campus and urban building types, capturing visual changes induced by diverse weather disturbances and thereby offering high visual diversity and environmental complexity. All images are standardized to a resolution of 1024 × 1024. The weather-augmented samples, as shown in Figure A1, are primarily designed to support systematic evaluation and comparative analysis of geo-localization model robustness and generalization under challenging weather conditions.
